# An integrative approach to discovering cryptic species within the *Bemisia tabaci* whitefly species complex

**DOI:** 10.1038/s41598-018-29305-w

**Published:** 2018-07-18

**Authors:** Soňa Vyskočilová, Wee Tek Tay, Sharon van Brunschot, Susan Seal, John Colvin

**Affiliations:** 1 Natural Resources Institute, University of Greenwich, Central Avenue, Chatham Maritime, ME4 4TB United Kingdom; 2 CSIRO Black Mountain Laboratories, Clunies Ross Street, ACT 2601 Canberra, Australia; 3 School of Biological Sciences, The University of Queensland, St Lucia, Queensland 4072 Australia

## Abstract

*Bemisia tabaci* is a cryptic whitefly-species complex that includes some of the most damaging pests and plant-virus vectors of a diverse range of food and fibre crops worldwide. We combine experimental evidence of: (i) differences in reproductive compatibility, (ii) hybrid verification using a specific nuclear DNA marker and hybrid fertility confirmation and (iii) high-throughput sequencing-derived mitogenomes, to show that the “Mediterranean” (MED) *B*. *tabaci* comprises at least two distinct biological species; the globally invasive MED from the Mediterranean Basin and the “African silver-leafing” (ASL) from sub-Saharan Africa, which has no associated invasion records. We demonstrate that, contrary to its common name, the “ASL” does not induce squash silver-leafing symptoms and show that species delimitation based on the widely applied 3.5% partial mtCOI gene sequence divergence threshold produces discordant results, depending on the mtCOI region selected. Of the 292 published mtCOI sequences from MED/ASL groups, 158 (54%) are low quality and/or potential pseudogenes. We demonstrate fundamental deficiencies in delimiting cryptic *B*. *tabaci* species, based solely on partial sequences of a mitochondrial barcoding gene. We advocate an integrative approach to reveal the true species richness within cryptic species complexes, which is integral to the deployment of effective pest and disease management strategies.

## Introduction

Accurate species identification underpins our understanding of biodiversity and enables clear scientific communication. In addition, if the organism is a pest having a negative impact on humans or the environment, it is essential for developing targeted and effective pest management strategies. Adequate species identification, however, is often difficult, especially when the organism belongs to a complex of cryptic species that cannot be distinguished by morphology alone. In such cases, it is logical to study populations of such organisms using the biological species concept. This concept defines a species as a group of individuals that can breed together (panmixis), but cannot with other groups, *i*.*e*. the group is isolated reproductively and genetically from other groups^1,2^. Evidence of both reproductive and genetic isolation is necessary for practical and accurate species identification while providing the opportunity to develop species-specific molecular markers. Ideally, an integrated approach involving the study of multiple factors (reproductive compatibility, morphology, ethology, ecology and molecular markers) is required to solve the problem^3,4^.

A popular and widely used technique for species assignment is DNA barcoding^5^. The process involves sequencing of a standardised region in the organism’s DNA and comparing it to an established ‘DNA Barcode’ database to enable species identity confirmation. For the Insecta, the commonly used region for DNA barcoding is a partial sequence of the mitochondrial cytochrome oxidase I (mtCOI) gene. Although this approach is attractive, simple and relatively fast, it has some fundamental problems, including: (i) the overlap between intra- and inter-species sequence divergences^6,7^, (ii) the large number of misidentified and erroneous sequences in online databases^8–10^, (iii) the arbitrary approach to selecting a ‘preferred’ barcoding gene region, *i*.*e*. either the 5′ region of mtCOI^5^ or its 3′ region^11^, and (iv) difficulties differentiating nuclear mitochondrial DNA segments (NUMTs) from genes in mitochondrial DNA (mtDNA)^12^.

The practice of submitting DNA sequences generated for barcoding purposes to public data repositories such as GenBank has made them readily available for further analyses including phylogenetic analyses. That is the case for the *Bemisia tabaci* cryptic species complex of whiteflies (Hemiptera: Aleyrodidae), some of whose members are regarded to be amongst the worst invasive pests and plant-virus vectors^13,14^. A considerable effort, therefore, has already been invested in attempting to resolve the systematics of this species group. The former system of classifying *B*. *tabaci* populations into biotypes and host races based on various biological and biochemical markers has been superseded by molecular analyses based on the 657 bp partial sequence of the 3′ end of the mtCOI gene^11,15–17^. In addition, a genetic distance threshold of 3.5% was identified based on a gap in the distribution of pairwise sequence divergences amongst unique mtCOI partial sequences of *B*. *tabaci*^16^. In subsequent analyses, patterns of clusters of putative species were recognised which could be defined by sequence divergence equal to or higher than 3.5%. Mating experiments among some of these phylogenetic species have reported either complete or partial reproductive isolation^18–21^.

At least 39 putative species have now been proposed in Bayesian and maximum likelihood phylogenetic analyses of the mtCOI sequenced from *B*. *tabaci* populations collected worldwide^7,16,22–29^. Of these, two species are especially of significant economic importance as highly invasive pests: Mediterranean (MED) and Middle East-Asia Minor 1 (MEAM1)^14^. These two species form the “Africa/Middle East/Asia Minor” clade together with the non-invasive Indian Ocean (IO) species, which was found to have asymmetric mating interactions with MEAM1 while showing 7–8% divergence at the mtCOI level^30^. Until recently, a Middle East-Asia Minor 2 (MEAM2) species also formed part of the clade, but its species status was based exclusively on pseudogene sequences originating from MEAM1 nuclear DNA^12^. A “MEAM2” population was recently reported from Uganda^31^, however, this is a new putative species that was named on the basis of 97.1% similarity (*i*.*e*. within the 3.5% species delimitation boundary) to a published MEAM2 pseudogene sequence and should not be confused with the MEAM2 pseudogene clade^12^.

The mtCOI partial sequence of MED corresponded to a syntype of *Aleurodes tabaci* originally collected by Gennadius in 1889, which indicates that MED represents the original *B*. *tabaci*^32^. MEAM1 was controversially named *B*. *argentifolii*^33^ due to its ability to induce silver-leafing symptoms when feeding on squash^34^. However, this trait was later also observed in IO^35^, and reportedly also in the “African silver-leafing” group of MED^14–16^.

MED was proposed to be a single species after grouping multiple biotypes (Q, J, L and “sub-Saharan African silver-leafing”), based on the 3.5% species boundary threshold^16^. An increased availability of new mtCOI sequences, however, led to the distinction of two subclades that were called Q1 and Q2^36^, or MedBasin1 and MedBasin2^37^, followed by further subdivision into four subclades (Q1, Q2, Q3, and African silver-leafing (ASL)^38^) or five subclades called Q1–Q5^39^, without an indication of how the group names corresponded to those previously published. Microsatellite analyses of sympatric field populations suggested that Q1 and Q2 were reproductively compatible^40–42^, while evidence of gene flow was not detected between Q1 and ASL in the field^43^, suggesting that the ASL population is a reproductively isolated entity. However, the model-based Bayesian methods for detecting hybrids that were implemented in the microsatellite field studies are biased towards the detection of F_1_ hybrids^44^. To ascertain reproductive compatibility between populations it is important to test the fertility of F_1_ hybrids.

In this study, we tested the hypothesis that the MED putative species (as described in^16,17,38,39^) constitutes a single biological species. We performed two-generational reciprocal crossing experiments in laboratory conditions associated with nuclear-gene based molecular markers to verify hybrid progeny. We then contrasted our findings with the mtCOI-based classification of MED and compared the sequence divergence among two different barcoding regions and 15 mitochondrial genes. Full mitogenomes were assembled from high-throughput sequencing (HTS) data, which enabled distinction between true mtDNA sequences and potential nuclear pseudogenes in the MED mtCOI datasets.

## Results

### F_1_ and F_2_ reciprocal crossing experiments

The lack of females in the F_1_ generation from reciprocal crosses revealed that the ASL population from Uganda was incompatible with both Q1 from Spain and Q2 from Israel. Unlike the control crosses that produced 31.4–41.8% females in the progeny, the reciprocal crosses involving ASL resulted in male offspring only (Table 1). Reciprocal crosses between Q1 and Q2, however, resulted in female and male offspring in both directions of the cross. Mean counts of F_1_ adults in these successful crosses, as well as proportions of females, were not significantly different from the controls (Table 1, Supplementary Table 1).

**Table 1:** Means and standard errors from reciprocal crossing experiments among the Spanish (Q1), Israeli (Q2) and Ugandan (ASL) populations of the MED putative species (*sensu* Dinsdale *et al*.^16^). Different superscript letters indicate statistically significant differences (P < 0.05) between crosses (Tukey’s test). The lowercase superscript letters relate to the multiple comparison of all 13 types of crosses, whereas the capital letters relate to a separate comparison including only crosses between Q1 and Q2.

Cross (1♀ × 3♂)	n	Mean no. of progeny	Mean no. of females	Mean % females
**Controls**				
♀Q1 × ♂Q1	9	21.8 ± 4.5^ad,AC^	9.1 ± 2.5	41.8 ± 8.2^ab,AB^
♀Q2 × ♂Q2	7	22.6 ± 5.3^ad,AC^	8.1 ± 2.6	36.1 ± 8.8^ab,AB^
♀ASL × ♂ASL	10	42.7 ± 8.2^d^	13.4 ± 3.5	31.4 ± 5.2^a^
**Reciprocal (F**_**1**_ **)**				
♀Q1 × ♂ASL	12	25.9 ± 4.6^bd^	0.0 ± 0.0	0.0 ± 0.0
♀ASL × ♂Q1	5	22.6 ± 6.3^ad^	0.0 ± 0.0	0.0 ± 0.0
♀Q2 × ♂ASL	13	17.4 ± 3.1^abc^	0.0 ± 0.0	0.0 ± 0.0
♀ASL × ♂Q2	7	20.1 ± 4.8^ad^	0.0 ± 0.0	0.0 ± 0.0
♀Q1 × ♂Q2	7	36.6 ± 8.4^cd,BC^	13.4 ± 4.2	36.7 ± 7.0^ac,A^
♀Q2 × ♂Q1	6	32.8 ± 8.2^bd,BC^	14.8 ± 4.9	45.2 ± 8.2^ab,AB^
**Reciprocal (F**_**2**_ **)**				
♀(♀Q1 × ♂Q2) × ♂Q1	12	42.2 ± 7.4^d,C^	25.1 ± 5.8	59.5 ± 5.1^bc,AB^
♀(♀Q1 × ♂Q2) × ♂Q2	11	41.1 ± 7.5^d,C^	26.8 ± 6.4	65.3 ± 5.2^b,B^
♀(♀Q2 × ♂Q1) × ♂Q1	7	11.7 ± 2.9^ab,AB^	9.6 ± 3.0	83.8 ± 9.6^bc,AB^
♀(♀Q2 × ♂Q1) × ♂Q2	5	6.8 ± 2.1^a,A^	6.0 ± 2.3	93.8 ± 9.9^ab,AB^

Fertility of the F_1_ hybrid females resulting from crosses between Q1 and Q2 was further investigated by backcrossing them with males of both parental types. In all four combinations, female offspring were produced (Table 1). The F_2_ females were present in 33 out of 35 replicates of all four combinations; the only two exceptions with purely male offspring occurred in the ♀ (♀Q2 × ♂Q1) × ♂Q2 cross.

There were, however, significant differences in the F_2_ offspring counts, depending on the direction of the cross (Table 1, Supplementary Table 2). The offspring of (♀Q2 × ♂Q1) females backcrossed with Q2 males was 6–6.2 times smaller than the one produced by (♀Q1 × ♂Q2) females backcrossed to males of either parental type, which was a highly significant difference (P < 0.001). The offspring of (♀Q2 × ♂Q1) females crossed with Q1 males was 3.5–3.6 times smaller than that of (♀Q1 × ♂Q2) females backcrossed to males of either parental type, which was also significantly different (P < 0.05).

The proportion of females also differed between F_1_ and F_2_ generations and between the directions of the cross. In controls and F_1_ reciprocal crosses of Q1 and Q2, the female percentage ranged from 36.1% to 45.2%. However, backcrossing the hybrid F_1_ females resulted in female-biased progeny with 59.5–93.8% F_2_ females. Within F_2_ crosses, there was a stronger bias (albeit non-significant) toward female offspring produced by hybrid females with a Q2 maternal origin (83.8% and 93.8% of F_2_ females) compared to their counterparts with a Q1 maternal background (59.5% and 65.3%).

### F_1_ hybrid verification by nuclear *GC1* marker

**Figure 1:**
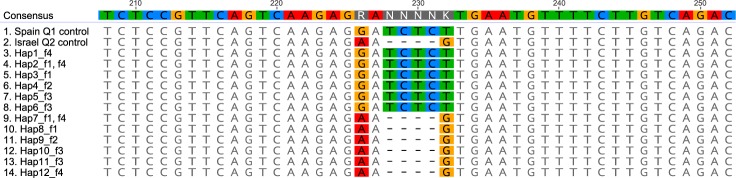
Molecular proof of parental origin of hybrid F_1_ females. Spain Q1 control and Israel Q2 control show population-specific polymorphisms: 4 bp TCTC INDEL and SNPs at positions 226 (Q1: ‘G’, Q2: ‘A’) and 232 (Q1: ‘T’, Q2: ‘G’). Unique haplotypes of the *GC1* marker (Hap1–Hap12) from four F_1_ females produced in reciprocal crosses between ♀Q1 × ♂Q2 (f1, f2) and ♀Q2 × ♂Q1 (f3, f4) were aligned against *GC1* sequences from females produced in control crosses (Q1 control, Q2 control). The presence of both Spain Q1- and Israel Q2-specific sequences in all four females confirm that the F_1_ females were hybrids between these two MED populations.

The parental origin of F_1_ females from the Q1 and Q2 cross was analysed by Sanger sequencing of the *GC1* nuclear DNA marker. Its sequence enabled the Q1 and Q2 populations to be distinguished based on a 4 bp insertion/deletion (INDEL) and two single nucleotide polymorphisms (SNPs) (Fig. 1). Analysis of trace files from direct Sanger sequencing of the *GC1* PCR product revealed heterozygous sequences in the F_1_ females, compared to the homozygous ones from control individuals. The heterozygosity was demonstrated as mixed traces downstream from the INDEL site.

The mixed sequences of *GC1* marker from four F_1_ females (f1 and f2 from ♀Q1 × ♂Q2; f3 and f4 from ♀Q2 × ♂Q1) were separated by molecular cloning and Sanger sequencing. The final sequence alignment of unique *GC1* haplotypes showed that each of the four F_1_ females contained sequences specific for both Q1 and Q2 populations (Fig. 1), confirming their hybrid status.

### Pseudogene identification

We applied four criteria in analysing published partial mtCOI sequences of five MED groups in order to detect potential pseudogene sequences. The presence of INDELs (criterion i) and significant amino acid changes (criterion iv) occurred in all five groups (Table 2). No premature stop codons (criterion ii) were detected, with the exception of clusters of non-synonymous mutations (criterion iii) found in Q1 and Q2 haplotypes.

**Table 2:** Summary of analysis of partial mtCOI sequences from GenBank against HTS-derived mitogenomes from this study. Numbers of haplotypes failing each criterion are shown in columns.

MED group	Total haplotypes	(i) INDEL	(ii) STOP	(iii) Polymorphisms	(iv) AA change^a^	Potential NUMTs	
						Total	%
Q1^b,c^	212	67	0	25	47	116	54.7%
Q2^b,c^	37	9	0	4	10	20	54.1%
Q3^c^	2	0	0	0	1	1	50.0%
Q3^b^ (=Q5^c^)	8	1	0	0	3	4	50.0%
ASL^b^ (=Q4^c^)	33	7	0	0	10	17	51.5%

^a^AA = amino acid. Only sequences without INDELs were analysed at the amino acid level. Only amino acid changes in sites conserved across all reference *Bemisia* species were considered.

^b^Naming system of Gueguen *et al*.^38^.

^c^Naming system of Chu *et al*.^39^.

Within the Q1 dataset, 67 (31.6%) of the 212 unique haplotypes failed criterion (i), out of which 12 overlapped with criterion (iii) (Supplementary Table 3). A particularly common INDEL was deletion of “A” at position 750 or 751 that occurred in 17 and 25 haplotypes, respectively. Criterion (iii) excluded 25 Q1 haplotypes, out of which two contained 40–41 SNPs within 757–769 bp (5.18–5.33% of the sequence), 11 contained 9–116 bp long stretches of non-synonymous mutations at the beginning or the end of the sequence (Supplementary Figure 1) and another 12 were identical to accessions from a different *Bemisia* species or chloroplast genome sequences in BLAST search. Criterion (iv) failed 47 (22.2%) Q1 haplotypes based on the alignment with a reference set of COI protein sequences, which revealed amino acid changes in positions that are fully conserved across the *B*. *tabaci* complex and the *Bemisia* “JpL” species.

The Q2 dataset also included haplotypes with INDELs (9/37, 24.3%), failing criterion (i). Four Q2 haplotypes were detected by criterion (iii). Three of them contained clusters with high density of non-synonymous mutations (20–21 bp on 5′ end and 87–89 bp at 3′ end; Supplementary Figure 2), including multiple INDELs. The fourth haplotype contained a high number of SNPs throughout the sequence (40 SNPs in 836 bp, 4.78% of the sequence). In addition, ten haplotypes (27.0%) showed non-synonymous substitutions resulting in amino acid changes in positions that were conserved across the reference set of protein sequences, failing criterion (iv).

The two haplotypes from Croatia, called Q3 in Chu *et al*.^39^, were 99.2–99.5% identical to the partial mtCOI of Israel Q2 and one of them contained a non-synonymous mutation in a conserved site. The group of haplotypes called Q3^38^ or Q5^39^, all from Burkina Faso, contained one haplotype with an INDEL and three haplotypes with one significant amino acid change. Finally, within the ASL^38^ or Q4^39^ group, 7/33 (21.2%) haplotypes contained INDELs and additional 10/33 (30.3%) haplotypes showed amino acid changes in conserved sites.

### mtCOI nucleotide divergence within the Africa/Middle East/Asia Minor clade

A sliding window analysis was performed to investigate and compare the level of nucleotide divergence in a 657 bp window across the full length of mtCOI gene (1,532 bp). Levels of nucleotide diversity in the 5′ and 3′ barcoding regions were compared.

**Figure 2:**
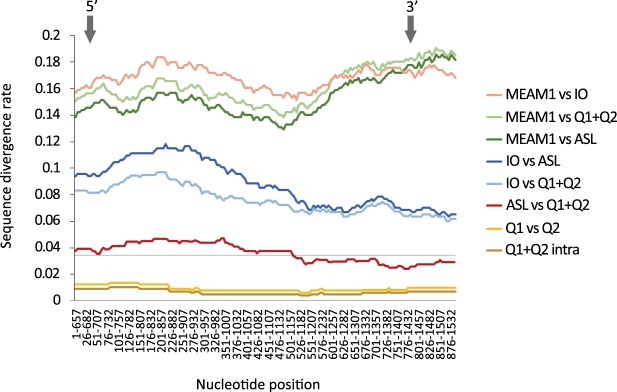
Pairwise sequence divergence across the full length of mtCOI gene (1,542 bp) from whiteflies of the Africa/Middle East/Asia Minor clade. The grey horizontal line marks the commonly applied 3.5% species boundary threshold^16^. The Q1 consists of two sequences (Spain Q1 and Burkina Faso Q1). Grey arrows indicate the position of 5′ and 3′ barcoding regions widely used to assist with species identification across taxa.

Pairwise comparisons among the putative species from Africa/Middle East/Asia Minor clade^16^ showed that the distribution of sequence divergence is not uniform across the gene length (Fig. 2). In comparisons Q1 + Q2 vs. ASL and MED vs. IO, the nucleotide divergence was higher towards the 5′ end of the mtCOI gene compared to the 3′ end. In contrast, in MEAM1 vs. all other samples the divergence rose even higher at the 3′ end compared to the 5′ end. In the 3′ region, therefore, the observed genetic distances between MEAM1 and MED or IO are higher than in the 5′ region.

The divergence between ASL and Q1 + Q2 groups ranged from 2.48 to 4.77% (Supplementary Table 4), depending on the chosen region of mtCOI gene. Thus, the divergence between the ASL population and other MEDs was either 1.02% below or 1.27% above the species delimitation boundary of 3.5% for the barcoding region selected for the *B*. *tabaci* species complex^16^. In contrast, the divergence between Q1 and Q2 populations showed a smaller variation in sequence divergence (0.61–1.38% depending on the region of mtCOI).

### Mitogenome divergence and phylogeny within members of *B*. *tabaci* species complex

The numbers of mapped Illumina HiSeq reads to the reference MED mitogenome JQ906700^45^ were 513,612 (Spain Q1), 249,142 (Israel Q2) and 149,343 (Uganda ASL), with mean base coverage 4606.5 ± 3502.6, 2231.5 ± 1576, and 1351 ± 666 reads, respectively.

**Figure 3:**
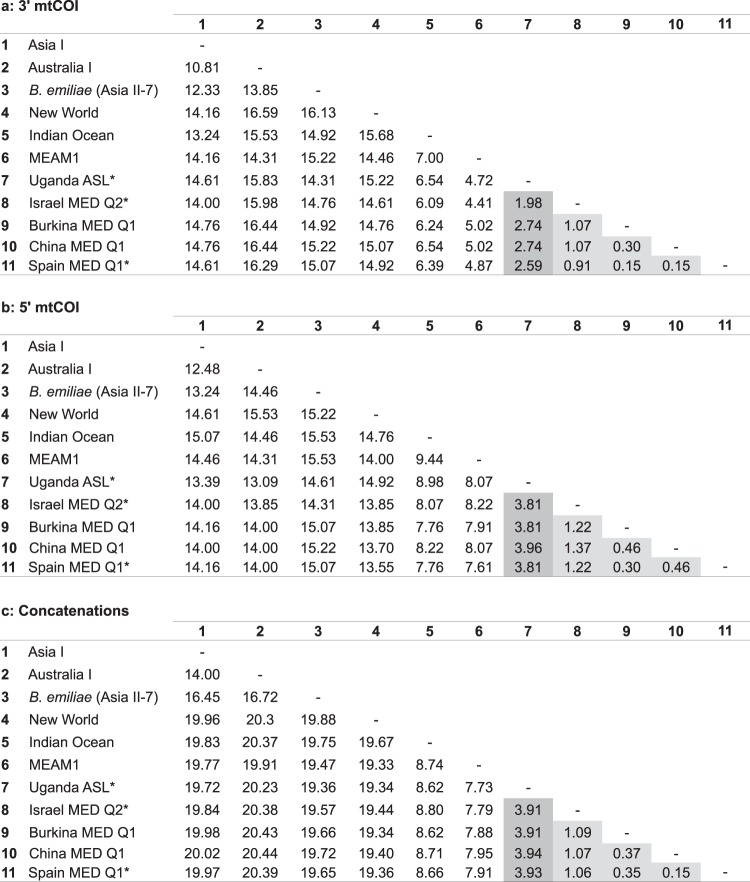
mtDNA sequence divergences amongst 11 species and populations of the *B*. *tabaci* complex. The regions compared were (**a**) the 3′ barcoding region of mtCOI used by the whitefly community (657 bp), (**b**) 5′ barcoding region of mtCOI used in the Barcode of Life (657 bp) and (**c**) the concatenations of 13 protein coding sequences and two rRNA genes in mtDNA (12,595 bp). The dark grey boxes highlight the divergences between Uganda ASL and MED populations, while divergences within Q1 and Q2 groups of MED are highlighted with light grey. Names with asterisks mark samples generated in this study.

The nucleotide divergence of mitochondrial DNA was compared amongst our populations and eight other species/populations of the *B*. *tabaci* species complex for three regions: (i) the 3′ mtCOI barcoding region (Fig. 3a), (ii) the 5′ mtCOI barcoding region (Fig. 3b) and (iii) the concatenation of 13 protein-coding sequences and 2 rRNA genes in the mitogenome (Fig. 3c).

The divergence within Q1 populations from Spain, China and Burkina Faso and Q2 from Israel remained low in all three regions, ranging from 0.15 to 1.37%. However, the divergence between the ASL population from Uganda and MED populations was below 3.5% only at the 3′ mtCOI barcoding region (range 1.98–2.74%), while at the 5′ the divergence rose to 3.81–3.96%. The divergence among the Q1, Q2, and ASL populations across 15 mitochondrial genes was more closely reflected in the 3′ region for Q1 and Q2 populations, while for the ASL population it was the 5′ region.

**Figure 4:**
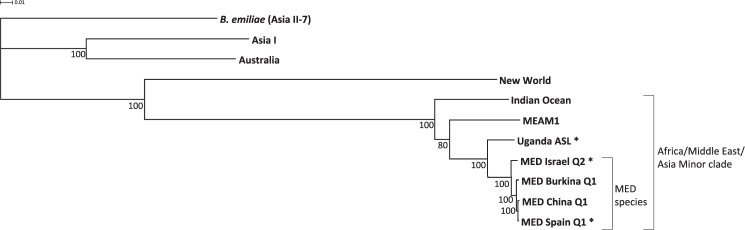
Unrooted maximum-likelihood phylogenetic tree inferred from 12,595 bp concatenations of 15 mitochondrial genes. The values below nodes show statistical support of Ultrafast Bootstrap. Names with asterisks mark samples generated in this study.

The relationships between MED populations, Uganda ASL population and six other species of *B*. *tabaci* complex were reconstructed in a phylogenetic analysis based on the concatenations of 15 mitochondrial genes (Fig. 4). The topologies of the resulting trees were identical in all three cases with or without data partitioning, however, the branch support was the highest after partitioning the data into 15 individual genes. The substitution models used for each partition were TPM2u + F + G4 (*atp6*), K3Pu + F + I (*atp8*), HKY + F + G4 (*cox1* and *cox2*), K3Pu + F + I + G4 (*cox3*), TIM + F + I + G4 (*cytb*), TPM3 + F + I (*nd1* and *rrnL*), TPM2u + F + I + G4 (*nd2*), TPM3 + F + G4 (*nd3*), TIM3 + F + I + G4 (*nd4*), TPM3u + F + I (*nd4l*), TIM3 + F + I + G4 (*nd5*), TPM2 + F + I (*nd6*), HKY + F + G4 (*rrnS*). The UltraFast bootstrap values for all branches were 80–100%, which unlike the normal bootstrap corresponds roughly to 80–100% probability that the clades are true^46^. Uganda ASL was placed outside the Q1 and Q2 clade with 100% bootstrap support, between MED and MEAM1 species.

### Biological and molecular assessment of the African silver-leafing population

**Figure 5:**
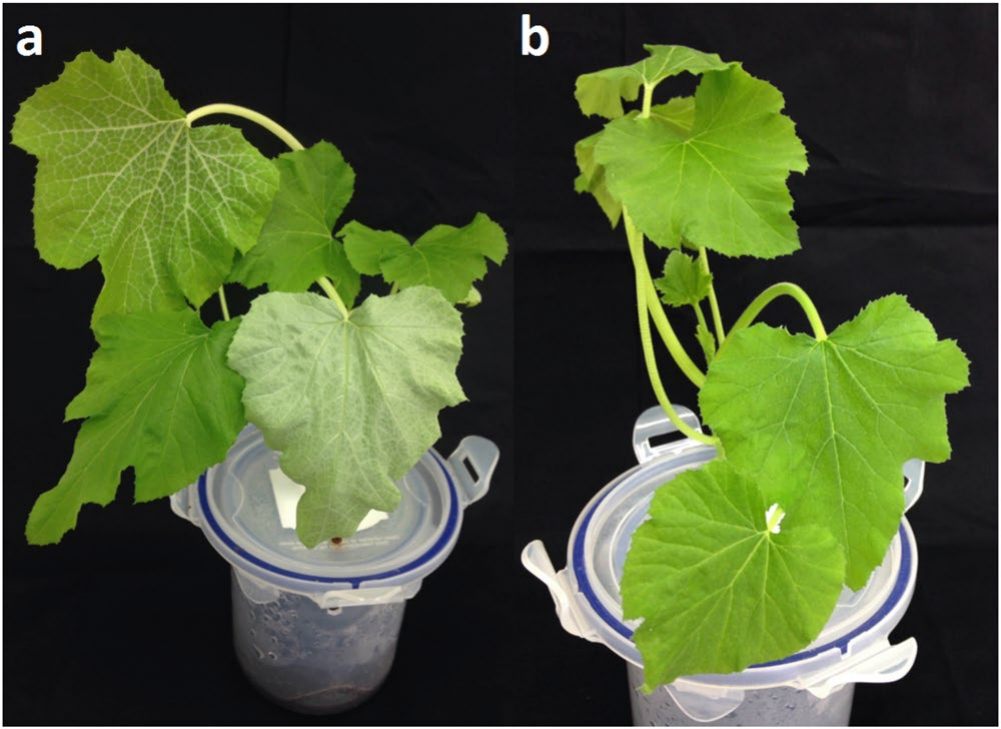
Squash plants (*Cucurbita pepo* ‘All green bush’) after feeding by: (**a**) MEAM1, and (**b**) Uganda “ASL”. Photographs were taken two weeks after infestation. The plant infested with MEAM1 showed leaf silvering symptoms, while the plant fed on by “ASL” population from Uganda did not show the symptoms. (Photograph by S. Vyskocilova).

The ability of the Ugandan “ASL” population to induce squash silver-leafing was tested in a bioassay with MEAM1 as a positive control. Squash plants infested with MEAM1 whiteflies developed silver-leafing symptoms within two weeks after whitefly infestation (Fig. 5a). The “ASL” population failed to induce the symptoms (Fig. 5b), even five weeks post-infestation.

The partial mtCOI sequences of our Uganda “ASL” population were compared with 27 published sequences of “sub-Saharan African silver-leafing”^15^, “okra biotype”^47^ and “Ug4”^48^ (Supplementary Table 5). After checking the sequence quality against the four criteria, 14 haplotypes were discarded as potential pseudogenes or errors. The remaining 13 haplotypes shared 98.87–100% similarity with Uganda “ASL”.

## Discussion

We demonstrate an integrative approach to resolving the systematics of a group of cryptic species, using the Mediterranean clade of the *B*. *tabaci* species complex as an example. The laboratory crossing experiments confirmed and expanded on the evidence of reproductive isolation between the sub-Saharan “ASL” population and the Q1 and Q2 MED whiteflies from the Mediterranean Basin^43^. Our backcrossing experiments confirmed the viability and fertility of Q1 × Q2 hybrids. A comparison of GenBank accessions of partial mtCOI sequences from different MED subclades with HTS-derived full mtCOI genes unravelled the scale of potential pseudogenes and/or low-quality sequences included in previous phylogenetic analyses. We also show that the “ASL” group is genetically distinct from the Q1 and Q2 MED groups, which are more closely related to one another. We also demonstrate that “ASL” does not induce silver-leafing in squash and so should be renamed.

The reproductive compatibility between Q1 and Q2 was previously suggested based on field population genetics studies^40–42^. Our study expanded on this evidence by laboratory crossing experiments in which the progeny counts and sex ratios could be observed and compared, as well as the viability and fertility of the hybrid progeny. In all four types of backcrossing, the F_1_ hybrids of Spain Q1 and Israel Q2 were fertile, regardless of which population was the source of maternal and paternal genetic background. In addition, for the first time, the parental origin of hybrid F_1_ females was verified by a molecular test based on a specifically selected nuclear marker.

Sequences of the cloned inserts of the *GC1* marker showed that the number of unique haplotypes present in three out of the four F_1_ females (f1, f3 and f4) tested, exceeded the theoretically possible number of alleles per individual. For example, two Q1-specific (Hap2 and Hap3) and two Q2-specific haplotypes (Hap7 and Hap8) were detected in the hybrid female f1, while in reality she would have received only one allele from each parent. The 12 *GC1* haplotypes differed from one another by 1–4 SNPs that occurred outside the diagnostic region shown in Fig. 1. These SNPs were probably errors introduced during the PCR amplification step by DreamTaq polymerase. The error rate of *Taq* DNA polymerases is 1–20 × 10^−5^ ^49–51^, which is 6- to 50-fold higher than more precise DNA polymerases such as *Pfu* or Phusion Hot Start^52^. Despite these introduced errors, the cloned sequences proved unequivocally that each hybrid female contained a copy from both parental types as shown by the INDEL region. This method of checking for genuine hybrid female progeny could be improved by using a high-fidelity DNA polymerase and a reduced number of PCR cycles for amplification of the marker sequence prior to cloning. Alternatively, the hybrids could be detected using microsatellite markers^40–42,53^ or evidence of hybridisation between species could also be demonstrated by using genome-wide SNPs^54^.

From the biological species concept definition, the Q1 and Q2 subclades belong to the same species. As the mtCOI of the original specimen of *B*. *tabaci* (Gennadius) from Greece corresponds to MED Q1^32^, we conclude that the Q1 and Q2 both belong to *B*. *tabaci sensu stricto*. These two groups are differentiated in allopatry, but retained the ability to interbreed when occurring sympatrically in the field. The gene flow between Q1 and Q2 MED populations was suggested to be asymmetric, because the nuclear background associated with Q1 individuals introgressed into, or completely replaced, the nuclear background of individuals with Q2 mitochondrial type, but not *vice versa*^41^. However, a different population genetic study based on microsatellites showed evidence for both directions of hybridisation^55^. Samples from France and Canary Islands had “Western Mediterranean” nuclear background and Q2 mitochondrial type, while sample from Greece had Q1-type mitochondria and “Eastern Mediterranean” DNA introgressed into its nuclear genome^55^. The interaction also did not appear asymmetric in Terraz *et al*.^40^, as the sympatric populations in France and Spain had homogeneous nuclear backgrounds regardless of the mitochondrial haplotype. In our study, no significant differences were observed between F_1_ progeny from the two directions of the cross. In the F_2_ generation, however, the fertility was unequal between hybrid females with different parental origin. Albeit the nuclear DNA of our samples was not studied, F_2_ females with Q1 mitochondria were more fecund than their Q2 counterparts, which could make hybrids with Q1 mitochondrial type more prevalent and thus more likely to be detected in the field.

We hypothesise that these mating interactions are influenced by secondary endosymbiotic bacteria. Some endosymbionts residing within insects are known to induce cytoplasmic incompatibility^56,57^ or affect the host’s fitness^58,59^. Cytoplasmic incompatibility could explain the mechanism behind the lack of gene flow between Q1 females and Q2 males in Gauthier *et al*.^41^. Alternatively, the asymmetry could be caused by the difference in fecundity between hybrids which emerged from the opposite directions of the same cross. The effect of endosymbiotic bacteria on the reproductive compatibility and fitness of MED whiteflies remains to be established.

The analysis of 292 unique mtCOI haplotypes deposited in GenBank as assigned to MED subclades Q1–Q5 and ASL^36–39^, revealed the extent of NUMTs or erroneous sequences included in previous molecular studies. In all five subclades, a half or more (50–55%) of the unique haplotypes contained mutations that are unlikely to occur in a functional protein-coding sequence. It is important to note that the FaBox tool assigned identical sequences with different lengths as unique haplotypes. Thus, the percentages in Table 2 probably do not reflect the frequency with which pseudogenes are picked up by the primers. The percentages are overestimated or underestimated, depending on whether it was pseudogenes/erroneous sequences, or mtDNA sequences predominantly deposited with unequal lengths.

The inclusion of NUMT sequences in phylogenetic analyses can overestimate the species richness^60^ and confound phylogenetic analyses^61,62^, as well as create confusion and inconclusive results in the research community. Pseudogene sequences have recently been shown to cause an overestimation of species richness within the Africa/Middle East/Asia Minor clade (the case of MEAM2^12^). This indicates that unrecognised NUMT sequences might commonly occur across multiple clades of the *B*. *tabaci* species complex and interfere with efforts to resolve its systematics. It is therefore important to recognise this issue and to analyse critically the quality of putative mitochondrial sequences amplified with universal primers. The pseudogene sequences are not immediately obvious if they do not contain stop codons or significant amino-acid changes within the analysed partial sequence. We emphasise the utility of HTS and acquiring mitochondrial sequences by assembly from a large number of reads. Such sequences can be then used as a reference for all the Sanger-sequenced marker sequences (e.g. from a field collection), to identify sequencing errors and potential pseudogenes and exclude them from downstream analyses.

The sub-Saharan ASL population is reproductively isolated from Q1 and Q2 groups as demonstrated by our laboratory crossing experiments and the field microsatellite study in Burkina Faso, where Q1 and ASL *B*. *tabaci* occurred in sympatry on the same host plants^43^. In addition, our phylogenetic tree shows the genetic distinctness of the ASL population from the MED Q1 and Q2 populations with a high statistical support. The tree also supports the consensus that Indian Ocean occupies a basal phylogenetic position within the Africa/Middle East/Asia Minor clade^7,14,16^.

The “Sub-Saharan Africa silver-leafing” clade was originally placed separately from the “Mediterranean invasive” clade in the first global Bayesian phylogenetic study by Boykin *et al*.^15^. These two clades, however, were later merged to form one “Mediterranean” putative species^16^. This placement was in accordance with the 3.5% species-level boundary in the 3′ barcoding region of mtCOI. We show here, however, that the identity values vary considerably across the full length of mtCOI gene. Results from the sliding window analysis demonstrate that the sequence divergence between ASL and Q1 + Q2 populations in a 657 bp long window ranged from 2.48 to 4.77% (a difference of 2.29%), depending on the region chosen for barcoding. Such variability is not apparent between Q1 and Q2, divergence of which remains low across the full gene length (0.61 to 1.38%, difference of 0.77%). A similar pattern was observed in the comparison at the mitogenome scale. Interestingly, both mtCOI barcoding regions quite accurately reflected the divergence across 15 mitochondrial genes, but each for a different group (3′ for Q1 and Q2; 5′ for ASL).

Our study also shows that the “African silver-leafing” population from Uganda does not induce silver-leafing. None of the plants infested with the ASL population developed silver-leafing symptoms five weeks after whitefly infestation, while the positive control with MEAM1 in our study and the one of Yokomi *et al*.^63^ showed symptoms within two weeks. This is not surprising, as the original paper by Sseruwagi *et al*.^48^ clearly stated that only groups Ug6 and Ug7 (corresponding to MEAM1 and IO, respectively) induced squash silver-leafing, while Ug4 (similar to “okra biotype” from Ivory Coast) did not. The “silver-leafing” group of MED was linked to the former J biotype^14^ reported in Nigeria, Ghana, Cameroon, Ivory Coast and Zimbabwe^64^. However, De la Rúa *et al*.^64^ did not study the capacity to induce silver-leafing and in earlier studies the J biotype failed to induce this phytotoxic disorder^65,66^. Despite all this evidence, multiple subsequent studies about African silver-leafing populations of MED referred either to Sseruwagi *et al*.^48^ or De la Rúa *et al*.^64^, or to other papers that have not demonstrated the silver-leafing capacity^14–16^. It is possible that the results of Sseruwagi *et al*.^48^ were misinterpreted due to the mixed “Ug4/Ug6” population reported to induce silver-leafing, and/or a confusion caused by the naming system in the study, and this misinterpretation was then cited in subsequent literature.

We propose that our “ASL” population from Uganda belongs to a non-MED species within the Africa/Middle East/Asia Minor clade. The 98.87–100% identity among 3′ mtCOI sequence across samples of “African silver-leafing”^15^, “okra biotype”^47,67^ and “Ug4”^48^ indicates that these groups might belong to the same species, but it remains to be confirmed. A list of these potential synonyms of the “ASL” species from previous literature is summarised in Supplementary Table 6. Similarly, the “Q3” population from Croatia might actually be part of Q2 together with our population from Israel based on their 99.2–99.5% identity. A mating study and ideally also a genome-wide SNP analysis would be needed to confirm these two results.

It is possible that “ASL” has already been described and was later synonymised with *B*. *tabaci* (Russell 1957). The synonymised species collected in sub-Saharan Africa include *B*. *gossypiperda var*. *mosaicivectura* Ghesquière (Congo, 1934), *B*. *vayssierei* Frappa (Madagascar, 1939), *B*. *goldingi* Corbett (Nigeria, 1935), *B*. *nigeriensis* Corbett (Nigeria, 1935) and *B*. *rhodesiaensis* Corbett (Rhodesia = Zimbabwe, 1936)^68^. DNA sequencing from museum syntypes of these species will be attempted in the near future to establish whether any of the above names apply to ASL/okra/Ug4. If not, a formal description of this new species will be required to accompany a new species name.

In conclusion, we gathered multiple lines of evidence that the MED species (*sensu* Dinsdale *et al*.^16^) comprises at least two biological species. One is the original *B*. *tabaci* (Gennadius), which includes the MED populations Q1 and Q2 from the Mediterranean Basin; the other is the sub-Saharan “MED ASL” species, awaiting a new binomial name. This division is based on the biological species concept, as “ASL” and MED are reproductively isolated both in the laboratory and in the field^43^. The reproductive compatibility of Q1 and Q2 was demonstrated in both directions, the fertility of F_1_ hybrids was verified and for the first time the combined genetic material from both parents in the F_1_ hybrids was confirmed using a specific nuclear DNA marker. Furthermore, by using HTS-derived mitogenomes, we were able to compare the genetic divergence among the MED populations and seven other *Bemisia* species, using true mitochondrial sequences (*i*.*e*. no pseudogenes) and multiple genes. This approach contrasts previous studies inferring phylogenetic relationships and sequence divergence among MED populations from 3′ partial mtCOI sequences, out of which 54% in the public databases contain errors and potentially are NUMTs.

The previous inclusion of the “ASL” population in the MED species demonstrates a fundamental problem with species delimitation based exclusively on an arbitrarily chosen barcoding sequence in mitochondrial DNA, because the results varied depending on the selected region of comparison. Furthermore, we showed a case of serial miscitation that led to the perpetuation of the erroneous assignment of the diagnostic trait of squash silver-leafing to the “ASL” species. Overall, we advocate the importance of an integrative approach to cryptic species identification and delimitation, most importantly including the biological species concept and using multiple genes or genomic data to infer *B*. *tabaci* phylogeny. With the increasing ease with which genome-wide SNPs can be obtained, combining nucleotide polymorphisms from both mitochondrial DNA and nuclear DNA genomes (e.g.^54^) will offer greater power for inference of *B*. *tabaci* cryptic species phylogenetic relationships. We also suggest that understanding the species diversity in pest-species complexes is the first step towards designing novel and targeted management strategies. It shall also bring consistency and clarity to communication within the scientific community, as well as to the wider public.

## Methods

### Insect rearing

*B*. *tabaci* colonies were reared on aubergine (*Solanum melongena* ‘Black Beauty’). Plants were grown from seeds in a whitefly-free room at 28 ± 2 °C, 50–60% relative humidity (r.h.) and a 14:10 L:D photoperiod. Whitefly colonies were maintained in rectangular 45 × 44 × 44 cm cages (BugDorm, US) at 28 ± 2°C, 30% r.h. and 14:10 L:D. Colonies were established from field populations collected in Spain (in 2013 from *Cucurbita* sp.; MED, Q1 group), Israel (in 2003 from *Gossypium hirsutum*; MED, Q2 group) and Uganda (in 2013 from *Abelmoschus esculentus*; MED, ASL group). The purity of core whitefly colonies was monitored periodically by sequencing of the partial mtCOI gene (detailed below).

### DNA extraction, molecular species identification and endosymbiont screening

Genomic DNA was extracted from adult whiteflies stored in 90% ethanol using the Chelex method^69^. At least five individual adults from each core colony were used for the species identity verification. Whiteflies were homogenised individually using sterile pestles in 50 µl of 10% Chelex® 100 Resin solution (Bio-Rad). The mixture was incubated at 56 °C for 20 min, subsequently at 95 °C for 5 min and centrifuged for 5 min at 13,500 rcf. The supernatant was used as a template for amplification by polymerase chain reaction (PCR).

Whitefly identification was based on the 3′ barcoding region of mtCOI gene. The segment was amplified and sequenced with primers 2195Bt and COI2-BtSh2^31^. PCRs (at 52 °C annealing temperature) were carried out using DreamTaq DNA polymerase (Thermo Scientific, UK) following the manufacturer’s instructions. The 867 bp amplicons were purified with reSource (Source Bioscience, UK) or GeneJET (Thermo Scientific, UK) PCR purification kits prior to Sanger sequencing by Source Bioscience (Nottingham, UK).

All DNA sequence analyses were carried out in Geneious version 10.0.8^70^, unless stated otherwise. The mtCOI sequences were trimmed to 657 bp, corresponding to nucleotide positions 782–1,439 of the complete mtCOI gene of MED^45^. Sequence-based comparisons were done using two reference datasets: (i) consensus sequences for 24 putative species of *B*. *tabaci*^16^ to verify MED species, and (ii) MED mtCOI haplotypes^38^ to place our populations in a recognised naming framework. In addition, the partial mtCOI sequences of Uganda ASL sample were compared to published sequences of “African silver-leafing”^15^, “okra biotype”^47,67^ and “Ug4”^48^ groups.

The presence/absence of the primary bacterial endosymbiont (*Portiera*) and five secondary endosymbionts (*Arsenophonus*, *Cardinium*, *Hamiltonella*, *Rickettsia* and *Wolbachia*) was tested by conventional PCR. Genus-specific primers targeting the 23 rDNA (*Hamiltonella*) or 16 S rDNA genes (the remaining bacteria) from Ghosh *et al*.^71^ were used. Total genomic DNA (extracted as above) from ten individual females per each colony was used as a template. Positive and negative controls were included in screening for each endosymbiont. PCR products were visualised by agarose gel electrophoresis, with results summarised in Supplementary Table 7.

### F_1_ and F_2_ reciprocal crossing experiments

Reproductive compatibility among the Q1, Q2 and ASL whitefly populations was determined by reciprocal crossing experiments. *B*. *tabaci* species produce males and females from unfertilised and fertilised eggs, respectively^72^. Gene flow between two populations, therefore, can be shown by the presence of female offspring.

All crosses were carried out using newly emerged virgin adults, isolated by excision as 4^th^ instar nymphs from a leaf and placed individually into glass tubes. Emerged adults were sexed visually on daily basis using a binocular stereomicroscope prior to mating experiments.

Control crosses were carried out with one female and three males from the same population with 8.7 replications on average. For the reciprocal crosses, the female and males originated from different populations and were used (average n = 8.3). The four adults were released onto aubergine plants in three to seven true-leaf stage, rooted in soil and enclosed in Lock&Lock whitefly-proof cages^73^ with additional side openings in the upper container covered by 160 µm nylon mesh. At least 24 h before introducing the adults, all leaves, except a fully expanded one, were removed to facilitate the contact between mating partners. Survival of the parental adults was monitored periodically and deceased males were replaced by new males from the respective colony. In the case of female death, the replicate was discarded. The parents were collected after seven days and stored in 90% ethanol at −20°C. All emerged adults of the F_1_ generation were collected and sexed. F_1_ females were stored for subsequent molecular analysis to verify their genetic make-up (detailed below).

The fertility of F_1_ hybrids was tested in a separate set of reciprocal crossing experiments. A female and three males per replicate (average n = 8.8) were used as above, but the F_1_ hybrid female nymphs were harvested from a plant onto which 20 + 20 virgin females and males from populations of interest were given the opportunity to reproduce for 14 days. Virgin hybrid females from both directions of the cross were then backcrossed with males from either parental population, resulting in four types of crosses to produce F_2_ generation. The parents were collected after seven days and progeny collected and sexed until all emerged.

Statistical analyses were performed using R^74^. Counts of offspring generated in crossing experiments were analysed by a generalised linear model with negative binomial error distribution and a log link using the MASS library^75^. For the proportion of female progeny, a generalized linear model with quasibinomial error distribution and logit link of the proportional data was used. Multiple comparisons of offspring counts and female proportions were performed by Tukey’s test^76^ using the multcomp package^77^ and significant differences were demonstrated by compact letter display. Reciprocal crosses that produced only male offspring were omitted from the multiple comparison. A separate analysis was also carried out with results from the F_1_ and F_2_ generations relevant only to the reproductively compatible populations.

### Molecular verification of F_1_ hybrid females

The parentage of female progeny was studied using a nuclear DNA marker. Mitochondrial DNA marker was not used due to its maternal mode of inheritance, rendering it ineffective for discerning the paternal origin of F_1_ females. We targeted nuclear genes encoding mitochondrial proteins, because of their potentially faster mutation rate^78^. Candidate genes were extracted from transcriptome sequences of adults from Spain MED Q1 and Israel MED Q2 populations (unpublished data) based on *Drosophila melanogaster* homologues (www.flybase.org) and searched for variable regions. The marker chosen for this study was a segment of 3′ untranslated region of the predicted Glutamate Carrier 1 (*GC1*) gene (annotated as Bta11593 in the genome of MEAM1^79^; 97% identity). Primers were developed in Primer 3 software^80^ (*GC1* primers; FWD3 5′-TGTTTTGTATTTTGATCTATTCA-3′, REV3 5′-CGAGGAGGAAATGTAAACAA-3′, annealing temperature 52 °C; expected amplicon size 770 bp).

The amplicon was sequenced from representative F_1_ females produced in reciprocal and control crosses. Trace files were analysed using the Pregap and Gap4 programs within the Staden molecular analysis software^81^. The *GC1* amplicons from F_1_ females were cloned into pGEM®-T Vector System I (Promega) with a 2:1 insert:vector ratio. Colonies of transformant *E*. *coli* cells JM109 were selected randomly and individually lysed in 200 µl sterile water by brief vortexing, then used as a template for PCR with vector-specific T7 and SP6 primers. Selected amplified inserts were sequenced at the ACRF Biomolecular Resource Facility at the Australian National University in Canberra and analysed as described above. The insert sequences were collapsed into unique haplotypes in FaBox DNA Collapser version 1.41^82^ and mapped to reference sequences from control crosses.

### High-throughput genome sequencing, quality control and mitogenome assembly

Genomic DNA from single male whiteflies representing each pure, inbred laboratory colony, was isolated using the silica spin-column method^83^ and quantified using a Qubit® 2.0 fluorometer (Invitrogen). Genomic DNA (_~_30 ng in 100 µl of TE buffer) was sheared for 10 min using an ultrasonic cleaner (VGT-1620QTD) and size-selected (300–500 bp) using Blue pippin (Sage Science). Sequencing libraries were prepared separately using each size-selected DNA pool using the NEBNext® Ultra DNA Library Prep Kit for Illumina® with NEBNext Multiplex Oligos for Illumina (New England BioLabs). Individual libraries were checked by capillary microchip electrophoresis (MultiNA, Shimadzu), pooled in equimolar amounts, and then purified using Agencourt AMPure XP Beads (Beckman Coulter). The multiplexed library was sequenced on a single lane of an Illumina HiSeq. 4000 platform (Novogene Bioinformatics Institute, Beijing, China) and 150 bp paired-end reads were generated.

Quality control was carried out in FastQC version 0.11.5 before and after trimming the reads with Skewer 0.2.2 version for Linux^84^. The reads trimmed with a quality threshold of 40 and minimum length of 18 bp were used in subsequent analyses. Draft mitogenomes of the Q1, Q2 and ASL males were assembled by iterative reference-guided assembly. The trimmed reads were mapped to the reference MED mitogenome JQ906700^45^ using Geneious 10.0.8 mapper set to medium-low sensitivity with up to 5 iterations. The draft mitogenomes were annotated in MITOS2^84^ and manually checked for start and stop codons in all protein-coding genes by translating into amino acid sequences using the invertebrate mtDNA genetic code. As the sequence was assembled from short reads, the accuracy of the low complexity intergenic region between *cox3* and *tRNA-Ile* represents only an estimate. Nevertheless, the lengths of these AT-rich regions among our mitogenomes (973 bp in Q1 and Q2, 971 bp in ASL) were similar to the one in the reference MED mitogenome JQ906700 (972 bp), which was obtained by long PCR and Sanger sequencing^45^.

### Pseudogene identification

Published partial mtCOI sequences of MED groups Q1, Q2, Q3, Q4, Q5 and ASL^36–39^ were downloaded from GenBank (accessed 14^th^ November 2017) and collapsed into unique haplotypes in FaBox (Supplementary Table 3). The haplotypes were then mapped to the full mtCOI genes extracted from the mitogenomes of Q1, Q2 and ASL from this study.

The criteria used to categorise a partial mtCOI sequence as a potential pseudogene included either: (i) the presence of INDELs, (ii) the presence of premature stop codons inside the protein-coding sequence, (iii) anomalous polymorphisms (*i*.*e*. clusters of non-synonymous mutations or haplotypes with an outlying sequence divergence compared to the intra-group divergence, and/or (iv) non-synonymous mutations resulting in amino-acid substitutions in positions conserved across other *Bemisia* species. For the fourth criterion, only sequences that passed the first two criteria were analysed. Such DNA sequences were translated and aligned by Geneious alignment tool using Blosum62 matrix to a reference set of mtCOI amino acid sequences translated from 12 mitogenomes: (1–3) populations from this study, (4) China MED Q1 (JQ906700)^45^, (5) Burkina Faso MED Q1 (KY951447), (6) Peru MEAM1 (KY951450), (7) Australia I (KY951541), (8) Indian Ocean (KY951448)^12^, (9) *B*. *emiliae* (former Asia II-7; KX714967), (10) *Bemisia* “JpL” (KX714968)^85^, (11) Asia I (KJ778614)^86^ and (12) New World (AY521259)^87^. The likelihood of the non-synonymous substitutions occurring in MED haplotypes was estimated by comparing the intra- and inter-species variability in respective triplet positions. Sequences with high nucleotide divergence from the group were compared against the NCBI database using BLASTn^88^ to investigate the origin of the sequence.

### Sliding window analysis

Intra- and inter-specific sequence divergence levels within the Africa/Middle East/Asia Minor clade^16^, excluding MEAM2 as a pseudogene artefact^12^, were compared across the full length of mtCOI gene (1,542 bp) in a sliding window analysis in DnaSP version 5.10.01^89^. The size of commonly used partial mtCOI sequence (657 bp) was used for the window size, sliding along the sequence in 5 bp steps while plotting the divergence (K-JC total) between the compared samples. Sequences included in the sliding window analysis were the three populations from this study, Burkina Faso MED Q1 (KY951447), Peru MEAM1 (KY951450) and Indian Ocean (KY951448)^12^.

### Mitogenome phylogeny and divergence

Concatenations of 13 protein-coding genes and two rRNA genes were generated from 11 mitogenomes listed above, excluding *Bemisia* “JpL” due to incomplete sequence data. The genes were orientated in 5′−3′ direction, individually aligned by MUSCLE alignment tool in Geneious and trimmed to the same length prior to being manually concatenated into 12,595 bp sequences. The multiple sequence alignment in fasta format was submitted to IQ-tree web server^90^ along with a manually created nexus file partitioning the data^91^. Three different partition schemes were analysed: (i) no data partitioning, (ii) partitioning the protein-coding genes into 1^st^ + 2^nd^ codon position, 3^rd^ codon position, and rRNA genes grouped in the third partition; and (iii) partitioning into 15 individual genes. No substitution saturation in the protein coding sequences was detected using DAMBE6^92^, indicating that all three codon positions could be used in the phylogenetic analysis. The best-fitting substitution model for each partition was identified using ModelFinder^93^. Ultrafast Bootstrap^94^ with 1,000 replications was performed and the tree was visualised in Dendroscope 3^95^.

### Squash silver-leafing bioassay

The capacity of Uganda ASL to induce the squash silver-leafing symptoms was tested by releasing 30–60 adults on squash plants *Cucurbita pepo* ‘All Green Bush’, individually enclosed in Lock&Lock cages in five replications. As a positive control, two replicates with 60 MEAM1 adults from Peru were set up using the same protocol. After two weeks the adults were collected and plants were visually assessed for leaf silvering symptoms, for up to five weeks post-infestation.

### Data availability

Draft mitogenomes assembled in this study are available under GenBank accession numbers MH205752 (Spain Q1), MH205753 (Israel Q2) and MH205754 (Uganda “ASL”). The partial 3′ mtCOI gene sequences (657 bp) of the whitefly colonies used in this study correspond to positions 782–1,439 of the draft mitogenomes.

Haplotypes of the *GC1* marker acquired from molecular cloning can be found under accessions MH205738–MH205749. Accessions for the *GC1* marker from Spain Q1 and Israel Q2 controls are MH205750 and MH205751, respectively.

## Electronic supplementary material

Supplementary Information
