# Supplementary material for: An integrative approach to discovering cryptic species within the Bemisia tabaci whitefly species complex
Source: Sci Rep. 2018 Jul 18;8:10886. doi: 10.1038/s41598-018-29305-w (PMC6052153; doi:10.1038/s41598-018-29305-w)
Supplement: Supplementary file 1 — Supplementary Information [file 41598_2018_29305_MOESM1_ESM.pdf]

An integrative approach to discovering cryptic species within the *Bemisia tabaci* whitefly species complex

Soňa Vyskočilová[1*], Wee Tek Tay[2], Sharon van Brunschot[1,3], Susan Seal[1], John Colvin[1]

[1] Natural Resources Institute, University of Greenwich, Central Avenue, Chatham Maritime, ME4 4TB, United Kingdom

[2] CSIRO Black Mountain Laboratories, Clunies Ross Street, ACT 2601, Australia

[3] School of Biological Sciences, The University of Queensland, St Lucia, Queensland, 4072, Australia

*Corresponding author, *s.vyskocilova@greenwich.ac.uk* / *s.vyskocilova@gmail.com*

Supplementary Table 1: P values from multiple comparison of progeny counts and female proportions from F_1 and F_2 crosses among Spain Q1, Israel Q2 and Uganda “ASL”. Key: n.c. = not compared due to the absence of females in the offspring, P<0.001 ‘***’, P<0.01 ‘**’, P<0.05 ‘*’.

Comparison	P values	
	**Progeny counts**	**Female proportion**
ASLxQ1 - ASL control	0.8001	n.c.
ASLxQ2 - ASL control	0.3890	n.c.
Q1 control - ASL control	0.4500	0.9717
Q1xASL - ASL control	0.7856	n.c.
Q1xQ2 - ASL control	1.0000	0.9994
Q1xQ2xQ1 - ASL control	1.0000	<0.01 **
Q1xQ2xQ2 - ASL control	1.0000	<0.01 ***
Q2 control - ASL control	0.6560	0.9999
Q2xASL - ASL control	0.0313 *	n.c.
Q2xQ1 - ASL control	0.9998	0.8727
Q2xQ1xQ1 - ASL control	<0.01 **	0.0309 *
Q2xQ1xQ2 - ASL control	<0.01 ***	0.3677
ASLxQ2 - ASLxQ1	1.0000	n.c.
Q1 control - ASLxQ1	1.0000	n.c.
Q1xASL - ASLxQ1	1.0000	n.c.
Q1xQ2 - ASLxQ1	0.9822	n.c.
Q1xQ2xQ1 - ASLxQ1	0.7927	n.c.
Q1xQ2xQ2 - ASLxQ1	0.8486	n.c.
Q2 control - ASLxQ1	1.0000	n.c.
Q2xASL - ASLxQ1	0.9999	n.c.
Q2xQ1 - ASLxQ1	0.9987	n.c.
Q2xQ1xQ1 - ASLxQ1	0.8628	n.c.
Q2xQ1xQ2 - ASLxQ1	0.1677	n.c.
Q1 control - ASLxQ2	1.0000	n.c.
Q1xASL - ASLxQ2	0.9998	n.c.
Q1xQ2 - ASLxQ2	0.8429	n.c.
Q1xQ2xQ1 - ASLxQ2	0.3625	n.c.
Q1xQ2xQ2 - ASLxQ2	0.4492	n.c.
Q2 control - ASLxQ2	1.0000	n.c.
Q2xASL - ASLxQ2	1.0000	n.c.
Q2xQ1 - ASLxQ2	0.9702	n.c.
Q2xQ1xQ1 - ASLxQ2	0.9328	n.c.
Q2xQ1xQ2 - ASLxQ2	0.2129	n.c.
Q1xASL - Q1 control	1.0000	n.c.
Q1xQ2 - Q1 control	0.9031	0.9999
Q1xQ2xQ1 - Q1 control	0.4141	0.6585
Q1xQ2xQ2 - Q1 control	0.5143	0.2847
Q2 control - Q1 control	1.0000	0.9999
Q2xASL - Q1 control	0.9998	n.c.
Q2xQ1 - Q1 control	0.9887	1.0000
Q2xQ1xQ1 - Q1 control	0.7787	0.2344
Q2xQ1xQ2 - Q1 control	0.0927	0.6377
Q1xQ2 - Q1xASL	0.9937	n.c.
Q1xQ2xQ1 - Q1xASL	0.7607	n.c.
Q1xQ2xQ2 - Q1xASL	0.8434	n.c.

Q2 control - Q1xASL	1.0000	n.c.
Q2xASL - Q1xASL	0.9293	n.c.
Q2xQ1 - Q1xASL	0.9999	n.c.
Q2xQ1xQ1 - Q1xASL	0.3010	n.c.
Q2xQ1xQ2 - Q1xASL	0.0116 *	n.c.
Q1xQ2xQ1 - Q1xQ2	1.0000	0.2007
Q1xQ2xQ2 - Q1xQ2	1.0000	0.0446 *
Q2 control - Q1xQ2	0.9618	1.0000
Q2xASL - Q1xQ2	0.3221	n.c.
Q2xQ1 - Q1xQ2	1.0000	0.9969
Q2xQ1xQ1 - Q1xQ2	0.0398 *	0.1044
Q2xQ1xQ2 - Q1xQ2	<0.01 ***	0.5087
Q1xQ2xQ2 - Q1xQ2xQ1	1.0000	0.9966
Q2 control - Q1xQ2xQ1	0.6336	0.3922
Q2xASL - Q1xQ2xQ1	0.0203 *	n.c.
Q2xQ1 - Q1xQ2xQ1	0.9998	0.8562
Q2xQ1xQ1 - Q1xQ2xQ1	<0.01 **	0.7868
Q2xQ1xQ2 - Q1xQ2xQ1	<0.01 ***	0.9227
Q2 control - Q1xQ2xQ2	0.7196	0.1399
Q2xASL - Q1xQ2xQ2	0.0381 *	n.c.
Q2xQ1 - Q1xQ2xQ2	1.0000	0.4879
Q2xQ1xQ1 - Q1xQ2xQ2	<0.01 **	0.9424
Q2xQ1xQ2 - Q1xQ2xQ2	<0.01 ***	0.9704
Q2xASL - Q2 control	0.9996	n.c.
Q2xQ1 - Q2 control	0.9970	0.9978
Q2xQ1xQ1 - Q2 control	0.7769	0.1352
Q2xQ1xQ2 - Q2 control	0.1011	0.5185
Q2xQ1 - Q2xASL	0.6686	n.c.
Q2xQ1xQ1 - Q2xASL	0.9854	n.c.
Q2xQ1xQ2 - Q2xASL	0.2937	n.c.
Q2xQ1xQ1 - Q2xQ1	0.1425	0.3315
Q2xQ1xQ2 - Q2xQ1	<0.01 **	0.7087
Q2xQ1xQ2 - Q2xQ1xQ1	0.9787	1.0000

Supplementary Table 2: P values from multiple comparison of progeny counts and female proportions from F_1 and F_2 crosses between Spain Q1 and Israel Q2 only. Key: P<0.001 '***', P<0.01 '**', P<0.05 '*'.

Comparison	P values	
	**Progeny counts**	**Female proportion**
Q1xQ2 - Q1 control	0.79854	0.9997
Q1xQ2xQ1 - Q1 control	0.34826	0.5898
Q1xQ2xQ2 - Q1 control	0.42791	0.2371
Q2 control - Q1 control	1.00000	0.9997
Q2xQ1 - Q1 control	0.94620	1.0000
Q2xQ1xQ1 - Q1 control	0.65150	0.1926
Q2xQ1xQ2 - Q1 control	0.08343	0.5680
Q1xQ2xQ1 - Q1xQ2	0.99984	0.1638
Q1xQ2xQ2 - Q1xQ2	0.99996	0.0347 *
Q2 control - Q1xQ2	0.88715	1.0000
Q2xQ1 - Q1xQ2	0.99999	0.9929
Q2xQ1xQ1 - Q1xQ2	0.04507 *	0.0831
Q2xQ1xQ2 - Q1xQ2	0.00185 **	0.4419
Q1xQ2xQ2 - Q1xQ2xQ1	1.00000	0.9923
Q2 control - Q1xQ2xQ1	0.52698	0.3339
Q2xQ1 - Q1xQ2xQ1	0.99563	0.8038
Q2xQ1xQ1 - Q1xQ2xQ1	0.00303 **	0.7256
Q2xQ1xQ2 - Q1xQ2xQ1	< 0.001 ***	0.8851
Q2 control - Q1xQ2xQ2	0.60325	0.1122
Q2xQ1 - Q1xQ2xQ2	0.99800	0.4225
Q2xQ1xQ1 - Q1xQ2xQ2	0.00522 **	0.9107
Q2xQ1xQ2 - Q1xQ2xQ2	< 0.001 ***	0.9498
Q2xQ1 - Q2 control	0.97568	0.9947
Q2xQ1xQ1 - Q2 control	0.65098	0.1087
Q2xQ1xQ2 - Q2 control	0.09120	0.4504
Q2xQ1xQ1 - Q2xQ1	0.13159	0.2786
Q2xQ1xQ2 - Q2xQ1	0.00758 **	0.6409
Q2xQ1xQ2 - Q2xQ1xQ1	0.91389	0.9999

Supplementary Table 3: List of unique published MED haplotypes (Q1, Q2, Q3/Q5, Q3 Croatia and ASL/Q4) and changes in DNA and amino acid sequences identified in them compared to the reference mtCOI from our HTS-derived mitogenomes. Positions in the last two columns refer to the nucleotide positions in which the change occurred. Haplotypes assigned to a particular criterion are colour-coded: criterion (i) INDELs, criterion (iii) outlying number of polymorphisms, criterion (iv) significant amino acid changes and an additional category sequence from a different species. Some haplotypes fall under two criterions. Premature STOP codons (criterion ii) were not detected outside clusters of non-synonymous mutations, which fall under criterion (iii). Key: n.s. = not studied, ins = insertion, del = deletion.

**Haplotype**	**Length (bp)**	**Accessions**	**DNA changes**	**Amino acid changes**
Q1_1	749	AM691051		
Q1_2	753	AM691057		
Q1_3	758	AM691058		
Q1_4	757	AM691080		
Q1_5	817	AM691084		
Q1_6	817	AM691052<br>DQ473394<br>EF694107<br>EF694109		
Q1_7	756	AM691068		
Q1_8	752	AM691071		
Q1_9	756	AM691054		
Q1_10	758	EU760734	751 del A	N.s.
Q1_11	791	EU760746	751 del A	N.s.
Q1_12	754	AM691056		1021 T->M
Q1_13	757	AM691072	40 SNPs	997 F->L
Q1_14	684	AM691067		
Q1_15	768	DQ365874	1512 del T	N.s.
Q1_16	768	DQ365875<br>DQ365876	1512 del T	N.s.
Q1_17	838	EU427719		709 G->C
Q1_18	807	EU427724		
Q1_19	776	AF342769	1522 del T	N.s.
Q1_20	729	EF398126<br>EF398114	1539 ins T	N.s.
Q1_21	803	EU760724	751 del A	N.s.
Q1_22	803	EU760738	751 del A	N.s.
Q1_23	805	EU760747	751 del A	N.s.
Q1_24	676	HM807533		
Q1_25	761	DQ365859	1512 del T	N.s.
Q1_26	809	FJ025793<br>FJ025794	1518 ins A	N.s.
Q1_27	810	FJ025796	1434 ins T, 1478 ins C	N.s.
Q1_28	808	FJ025797	1518 ins A	N.s.
Q1_29	809	FJ025795	738 ins G, 755 ins T, 765 ins T, 822 ins T, 1518 ins A	N.s.
Q1_30	817	HM590170	100% identical to *B. tabaci* Asia I KR020523, JN855568, HM590165	N.s.

			and KF790648 from India; HG918196, HG315654 and HF934996 from Pakistan; KJ778614 from Bangladesh	
Q1_31	778	AY057179	AY057179 = a cassava whitefly from Uganda	N.s.
Q1_32	777	AY057180	AY057180 = a cassava whitefly from Uganda<br>100% identical to KX570785 and AM040604 from Uganda; JQ286450 from Tanzania	N.s.
Q1_33	778	AY057169	AY057169 = a cassava whitefly from Uganda	N.s.
Q1_34	783	AY057170	802 ins T<br>AY057170 = a cassava whitefly from Uganda	N.s.
Q1_35	779	AY057171	AY057171 = a cassava whitefly from Uganda	N.s.
Q1_36	783	AY057163	AY057163 = a cassava whitefly from Uganda<br>100% identical to KX570846, KX570845, AM040603 and AF418669 from Uganda	N.s.
Q1_37	778	AY057162	AY057162 = a cassava whitefly from Malawi	N.s.
Q1_38	657	GU086333		
Q1_39	781	GQ139499<br>GQ139501<br>GQ139502		
Q1_40	781	GQ139500		787 L->W
Q1_41	781	GQ139503		
Q1_42	721	GQ139504		
Q1_43	817	GQ371165		1480 K->S
Q1_44	728	EF398122		
Q1_45	729	EF398116	1539 ins T	N.s.
Q1_46	615	EF398118		
Q1_47	720	HM586106		
Q1_48	649	GU168791<br>GU168792<br>GU168794<br>GU168795<br>GU168796<br>GU168797<br>GU168798<br>GU168799<br>GU168800		
Q1_49	649	GU168793		787 L->F
Q1_50	657	GU086329		
Q1_51	818	EU263633	1464 del C, 1483 del A	N.s.
Q1_52	831	EU263631		
Q1_53	831	EU263629		
Q1_54	844	EU192071	1539 ins T	N.s.

Q1_55	842	EU192072	1528 del A	N.s.
Q1_56	811	EU263630	Last 14 bp cluster of mutations, 1415 del T	N.s.
Q1_57	657	GU086332		
Q1_58	807	EU263626	Last 22 bp cluster of mutations, 766 del A	N.s.
Q1_59	816	EF667477		736 L->F
Q1_60	814	EF566760		
Q1_61	818	EF667474		
Q1_62	789	EF694104		
Q1_63	788	EF694105<br>EF694106<br>FJ375358<br>FJ375350<br>FJ375354<br>FJ375355<br>FJ375351<br>FJ375348<br>FJ375353<br>FJ375352<br>FJ375347<br>FJ375357<br>FJ375356<br>FJ375346		
Q1_64	759	HM137324		
Q1_65	759	HM137360		1477 W->C
Q1_66	759	HM137334		
Q1_67	859	HM597854	First 42 bp cluster of mutations, 763 ins G	N.s.
Q1_68	813	HM597855	751 del A	N.s.
Q1_69	836	HM597863		
Q1_70	836	HM597849	751 del A	N.s.
Q1_71	844	HM597865		
Q1_72	833	HM597869		
Q1_73	680	FJ594432<br>FJ594429<br>FJ594433<br>FJ594431<br>FJ594434<br>FJ594428<br>FJ594430		
Q1_74	836	HM597870<br>HM597871		
Q1_75	854	HM597852	First 27 bp cluster of mutations, 738 del A	N.s.
Q1_76	851	HM626150	751 del A, 1404 ins T	N.s.
Q1_77	850	HM626151	740 del C, 751 del A	N.s.
Q1_78	720	HM137321		1477 W->C
Q1_79	856	HM626152	First 17 bp cluster of mutations, 751 del A	N.s.
Q1_80	759	HM137331		
Q1_81	863	HM626153	First 29 bp cluster of mutations	748 A->G

				751 I->T<br>763 L->W
Q1_82	846	HM597864	751 del A	N.s.
Q1_83	837	HM597848	751 del A	N.s.
Q1_84	843	HM597850	751 del A	N.s.
Q1_85	835	HM597857		
Q1_86	855	HM597853	751 del A	N.s.
Q1_87	834	HM597868		
Q1_88	839	HM597847	751 del A	N.s.
Q1_89	788	FJ375349		1477 W->C
Q1_90	837	HM597851	751 del A	N.s.
Q1_91	835	HM597867		
Q1_92	839	HM597862		739 P->T
Q1_93	833	HM597859		754 V->A<br>769 S->I
Q1_94	838	HM597866		
Q1_95	871	HM802266		709 G->C
Q1_96	866	HM802267		709 G->C
Q1_97	870	HM802268		709 G->C
Q1_98	841	HM597856	751 del A	N.s.
Q1_99	736	FJ188504		
Q1_100	781	FJ188524		
Q1_101	773	FJ188507		
Q1_102	784	FJ188508		
Q1_103	784	FJ188552		
Q1_104	821	FJ188553		
Q1_105	814	EU427722		
Q1_106	1242	EU427723		
Q1_107	835	EF080823		709 G->C
Q1_108	829	EU427725	1512 del T	N.s.
Q1_109	769	FJ188615	41 SNPs	
Q1_110	545	JN966761		
Q1_111	687	DQ989547<br>DQ989548<br>DQ989549<br>DQ989550		
Q1_112	656	HM488309<br>HM488310<br>HM488311		
Q1_113	656	HM488312<br>HM488313<br>HM488314<br>HM488331	751 del A	N.s.
Q1_114	656	HM488315	751 del A	N.s.
Q1_115	656	HM488324<br>HM488327<br>HM488332<br>HM488338	751 del A	N.s.
Q1_116	658	HM488325	751 del A , 1378 ins T	N.s.

Q1_117	657	HM488326<br>HM488329<br>HM488330<br>HM488333<br>HM488334<br>HM488335<br>HM488336<br>HM488337<br>HM488339	751 del A	N.s.
Q1_118	657	HM488328<br>HM488328	751 del A , 1358 ins T	N.s.
Q1_119	748	DQ462583<br>DQ462584<br>DQ462585		
Q1_120	748	DQ462586		
Q1_121	676	HM807573<br>HM807574<br>HM807549<br>HM807580<br>HM807579		
Q1_122	777	AB204586<br>AB204587<br>AB204588		
Q1_123	777	AB204579		
Q1_124	796	EU760723	751 del A	N.s.
Q1_125	741	FJ766417		
Q1_126	816	AJ517769		1018 F->S
Q1_127	730	DQ174540		1186 F->S
Q1_128	779	AY057178	AY057178 = a cassava whitefly from Uganda	N.s.
Q1_129	730	DQ174539		1327 A->G<br>1399 F->S<br>1402 L->S<br>1414 L->F<br>1432 L->S<br>1453 L->P
Q1_130	799	AY057174	AY057174 = a sweet-potato whitefly from Uganda	N.s.
Q1_131	789	AM691053		
Q1_132	817	AM691050		
Q1_133	817	AM691055		937 P->L<br>1207 T->A
Q1_134	817	AM691063		910 T->I<br>1411 V->A
Q1_135	761	DQ365857<br>DQ365858<br>DQ365860<br>DQ365862<br>DQ365863<br>DQ365865<br>DQ365866<br>DQ365867<br>DQ365868	1512 del T	N.s.

		DQ365869<br>DQ365870		
Q1_136	761	DQ365871	1512 del T	N.s.
Q1_137	676	HM807578		
Q1_138	800	AY903578		892 D->G
Q1_139	684	DQ302946		
Q1_140	757	AM691069<br>AM691074		
Q1_141	759	AM691070		
Q1_142	757	AM691075		
Q1_143	762	AM691076		
Q1_144	752	AM691077		
Q1_145	758	AM691079		
Q1_146	761	AM691081		
Q1_147	750	AM691082		
Q1_148	724	AM691083		
Q1_149	801	EU760753	751 del A	N.s.
Q1_150	730	DQ174541		1453 L->P
Q1_151	657	GU086330		
Q1_152	761	DQ365856<br>DQ365861<br>DQ365864	1512 del T	N.s.
Q1_153	780	EU760736		
Q1_154	676	HM807571<br>HM807563<br>HM807560<br>HM807561<br>HM807550		
Q1_155	728	AY827614		766 I->M<br>865 H->P
Q1_156	739	AY827613		766 I->M<br>820 A->P
Q1_157	730	AY827612		862 H->P
Q1_158	739	AY827615		766 I->M<br>826 L->W<br>829 T->P<br>844 G->S<br>871 F->I<br>916 A->G<br>1078 S->P
Q1_159	728	EF398125		859 G->R
Q1_160	728	EF398121		862 H->Q
Q1_161	729	EF398117	1539 ins T	N.s.
Q1_162	728	EF398120		862 H->N<br>901 A->V
Q1_163	728	EF398115		
Q1_164	729	EF398123		
Q1_165	778	AF342773		1021 T->M
Q1_166	687	DQ989546		1021 T->M
Q1_167	822	EU099427	First 116 bp cluster of mutations,<br>750 del A	N.s.

Q1_168	789	DQ133378	First 8 bp cluster of mutations 750 del A, 765 del A, 1499 del T, 1510 del TT	N.s.
Q1_169	788	DQ133379	First 7 bp cluster of mutations 750 del A, 765 del A, 1501 del A, 1507 del G	N.s.
Q1_170	790	DQ133380	First 9 bp cluster of mutations 750 del A, 765 del A, 1501 del A, 1510 del TT	N.s.
Q1_171	806	EU760726	750 del A	N.s.
Q1_172	807	EU760730	750 del A	N.s.
Q1_173	812	EU760732	750 del A	N.s.
Q1_174	794	EU760722	1459 del TA	N.s.
Q1_175	811	EU760728	750 del A	N.s.
Q1_176	798	EU760729		
Q1_177	741	FJ766392<br>FJ766394<br>FJ766396<br>FJ766397<br>FJ766401<br>FJ766414<br>FJ766430<br>FJ766436<br>FJ766426<br>FJ766425		
Q1_178	741	FJ766381		
Q1_179	673	FJ766125	FJ766125 = AtpF gene in chloroplast DNA from *Cenchrus compressus*	N.s.
Q1_180	650	FJ766126	FJ766126 = AtpF gene in chloroplast DNA from *Phacelurus latifolius*	N.s.
Q1_181	676	HM807568<br>HM807566<br>HM807562<br>HM807551<br>HM807548<br>HM807543<br>HM807544<br>HM807545<br>HM807546<br>HM807538<br>HM807539		
Q1_182	776	AY057138		766 I->M<br>1270 F->C
Q1_183	831	EU192061	First 10 bp cluster of mutations 710 del GT, 750 ins GA, 750 del A, 1511 del T	N.s.
Q1_184	831	EU192049	First 10 bp cluster of mutations 710 del GT, 750 ins GA, 750 del A, 1511 del T	N.s.
Q1_185	657	GU086337		1258 V->G
Q1_186	545	JN966876		1258 V->G

Q1_187	657	GU086338		1258 V->E
Q1_188	657	GU086336		805 L->W<br>922 M->K<br>973 L->W<br>1258 V->E
Q1_189	657	GU086339		
Q1_190	817	EF694108		1078 S->F<br>1360 S->F
Q1_191	716	AM691064		1219 Y->H
Q1_192	818	AM176575		988 S->F
Q1_193	817	AM176574		1054 L->S<br>1447 F->L
Q1_194	817	AM176571		
Q1_195	817	AM180063		769 S->G<br>988 S->P<br>1045 M->T
Q1_196	759	HM137320		1219 Y->C<br>1477 W->L
Q1_197	759	HM137333		117 V->D
Q1_198	759	AM691059		
Q1_199	793	EU760743	750 del A	N.s.
Q1_200	805	EU760744	750 del A	N.s.
Q1_201	545	JN966877<br>JN966878<br>JN966879<br>JN966880		
Q1_202	755	AM691062		
Q1_203	795	EU760737	750 del A	N.s.
Q1_204	741	FJ766432<br>FJ766434		
Q1_205	807	EU760725	750 del A	N.s.
Q1_206	770	EU760721		
Q1_207	817	AM176573		1153 I->T
Q1_208	545	JN966871<br>JN966872<br>JN966873<br>JN966874<br>JN966875		1369 S->G
Q1_209	798	EU760755	750 del A	N.s.
Q1_210	801	EU760749	750 del A	N.s.
Q1_211	795	EU760761	1459 del T	N.s.
Q1_212	790	EU760740	750 del A, 1459 del T, 1483 ins A, 1512 ins T	N.s.
Q2_1	777	AF342776		1361 S->F
Q2_2	772	AM944348<br>AM944347		
Q2_3	501	AY518191	787 ins A, short sequence	N.s.
Q2_4	768	DQ365877	1512 del T	N.s.
Q2_5	747	DQ365878		
Q2_6	852	EF080821		709 G->C

Q2_7	729	EF398128	1539 ins T	N.s.
Q2_8	838	EU427721		709 G->C
Q2_9	836	EU760719	40 SNPs	997 F->L<br>1528 I->T
Q2_10	803	EU760751		
Q2_11	804	EU760754	751 del A	N.s.
Q2_12	797	EU760756	751 del A	N.s.
Q2_13	785	EU760757		
Q2_14	776	FJ998204	843 ins T, 957 del T, 1498 del C	N.s.
Q2_15	779	AY766372	798-818 cluster of mutations; 804 ins A, 1496 ins GGACT, 1515 ins C, last 87 bp cluster of mutations	N.s.
Q2_16	676	HM807534		
Q2_17	687	DQ989554<br>DQ989553		
Q2_18	777	AB297895<br>AB297896		
Q2_19	740	AY827617<br>AY827618		766 I->M<br>769 S->G
Q2_20	811	FJ188567		
Q2_21	837	FJ188539		709 G->C
Q2_22	800	FJ188558		
Q2_23	823	FJ188480		
Q2_24	822	FJ188481<br>FJ188559		
Q2_25	821	FJ188482		
Q2_26	745	FJ188483		
Q2_27	803	FJ188543		
Q2_28	802	FJ188541		
Q2_29	545	JN966827<br>JN966870		
Q2_30	740	AY827619		766 I->M<br>769 S->G<br>829 T->A<br>838 I->M<br>946 I->M
Q2_31	657	GU086331		1361 S->F
Q2_32	780	AY766371	798-816 cluster of mutations; 798 ins A, 1483 ins AC, last 89 bp cluster of mutations	N.s.
Q2_33	780	AY766370	798-819 cluster of mutations; 798 ins A, 1485 del T, last 88 bp cluster of mutations	N.s.
Q2_34	777	AB297897		1183 W->R<br>1468 H->R
Q2_35	777	AB297898		796 F->V<br>1297 G->E
Q2_36	727	EU760756		
Q2_37	715	EU760757		
Q3_Croatia_1	657	GU086334		1259 V->E

Q3_Croatia_2	657	GU086335		
Q3/Q5_1	741	FJ766382<br>FJ766386<br>FJ766387<br>FJ766395<br>FJ766409<br>FJ766424<br>FJ766427<br>FJ766417		
Q3/Q5_2	741	FJ766405<br>FJ766419		
Q3/Q5_3n	741	FJ766420<br>FJ766421<br>FJ766422<br>FJ766423<br>FJ766400<br>FJ766428		
Q3/Q5_4	741	FJ766429		1204 L->V
Q3/Q5_5	741	FJ766384		1204 L->V
Q3/Q5_6	737	FJ766431		1204 L->V
Q3/Q5_7	740	FJ766408	1515 del A	N.s.
Q3/Q5_8	741	FJ766385		
ASL/Q4_1	800	AY903549<br>AY903533<br>AY903555<br>AY903572<br>AY903574		
ASL/Q4_2	800	AY903532		
ASL/Q4_3	800	AY903556		
ASL/Q4_4	800	AY903551		1531 F->S<br>1393 I->M
ASL/Q4_5	800	AY903566<br>AY903546<br>AY903545		
ASL/Q4_6	800	AY903550	943 ins A	N.s.
ASL/Q4_7	749	AY827606		775 E->K
ASL/Q4_8	800	AY903564	1538 del T	N.s.
ASL/Q4_9	800	AY903565	15 SNPs	790 E->K<br>805 L->V<br>853 V->G<br>859 G->S<br>862 H->P<br>898 R->P<br>1027 F->I<br>1114 V->G<br>1117 V->G<br>1129 H->P
ASL/Q4_10	800	AY903552	949 ins T	N.s.
ASL/Q4_11	800	AY903529	1539 ins T	N.s.
ASL/Q4_12	800	AY903573		1003 P->A<br>1360 S->F<br>1498 L->F

ASL/Q4_13	800	AY903531		
ASL/Q4_14	800	AY903541<br>AY903540		
ASL/Q4_15	800	AY903534	1538 del T	N.s.
ASL/Q4_16	800	AY903535	1083 ins C	N.s.
ASL/Q4_17	740	AY827590		
ASL/Q4_18	740	AY827588		
ASL/Q4_19	741	FJ766388<br>FJ766390<br>FJ766391<br>FJ766393<br>FJ766398<br>FJ766410<br>FJ766413<br>FJ766415		
ASL/Q4_20	741	FJ766389		1468 H->Y
ASL/Q4_21	741	FJ766435		
ASL/Q4_22	740	AY827587		
ASL/Q4_23	739	AY827582	19 SNPs	763 L->C<br>775 E->K<br>790 E->A<br>817 Y->M<br>1414 L->S<br>1438 L->W<br>1486 N->I<br>1489 K->N
ASL/Q4_24	740	AY827579		1444 S->K
ASL/Q4_25	740	AY827589		
ASL/Q4_26	709	AY827581		1249 F->C<br>1372 I->N<br>1438 L->W
ASL/Q4_27	740	AY827580		1249 F->C<br>1405 F->L<br>1468 H->L<br>1474 E->D
ASL/Q4_28	741	FJ766418<br>FJ766399<br>FJ766402<br>FJ766403<br>FJ766404<br>FJ766406<br>FJ766411<br>FJ766407<br>FJ766412<br>FJ766416		
ASL/Q4_29	741	FJ766433<br>FJ766437		
ASL/Q4_30	778	AY057136		1519 L->I<br>1525 L->W<br>1528 I->S
ASL/Q4_31	741	FJ766383		
ASL/Q4_32	808	EU760731	751 del A	N.s.
ASL/Q4_33	783	EU760758		

Supplementary Figure 1: Alignments of eleven MED Q1 haplotypes containing clusters of non-synonymous mutations and INDELs (**a**) at 5' end and (**b**) at 3' end of the partial mtCOI sequence.

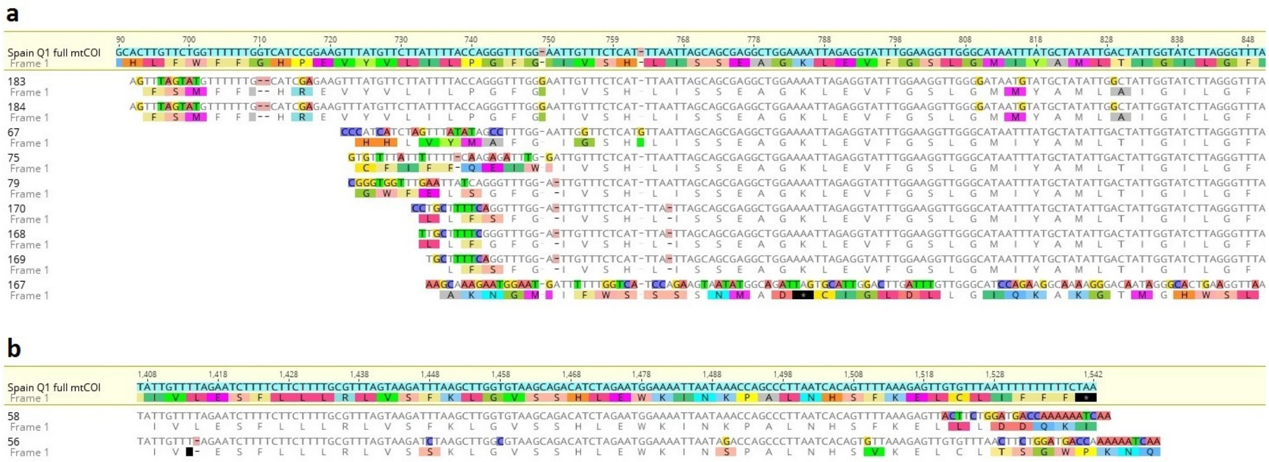

Supplementary Figure 2: Alignments of three MED Q2 haplotypes containing clusters of non-synonymous mutations and INDELs (**a**) at 5' end and (**b**) at 3' end of the partial mtCOI sequence.

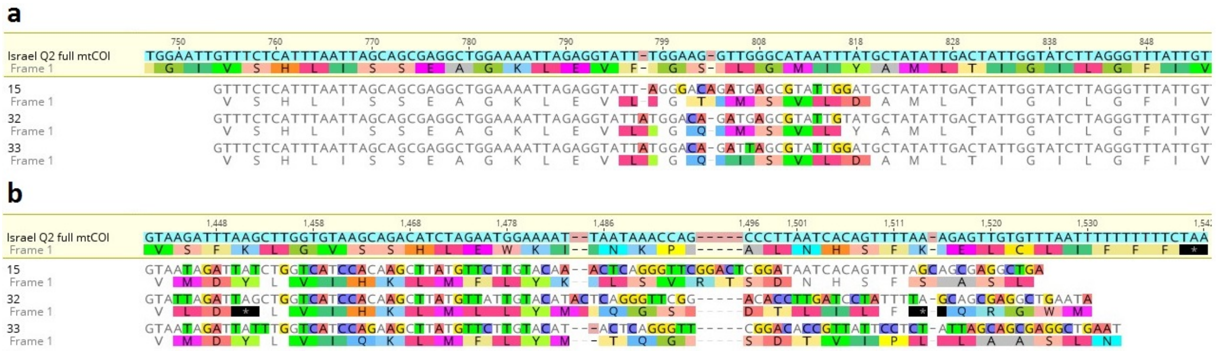

Supplementary Table 4: Results from the sliding window analysis of sequence divergence across the full length of mtCOI gene (1,542 bp) from whiteflies of the Africa/Middle East/Asia Minor clade. The positions or ranges of positions are indicated in parentheses for each minimum and maximum observed divergence.

Samples compared	Minimum K (JC-total) (positions)	Maximum K (JC-total) (positions)	K (JC-Total)	Notes
**Q1+Q2 intra**	0.00406 (526-1182, … 546-1202)	0.01015 (81-737, … 146-802)	Pi (JC-Total): 0.00739	Q1+Q2 = Spain + Burkina + Israel.
**Q1 vs. Q2**	0.00611 (526-1182 … 546-1202)	0.01383 (81-737, … 146-802)	0.01012	Q1 = Spain + Burkina. Q2 = Israel
**ASL vs. Q1+Q2**	0.02476 (781-1437, … 791-1447)	0.04765 (346-1002, 351-1007)	0.03564	
**ASL vs. MEAM1**	0.12924 (486-1142)	0.18556 (841-1497, … 876-1532)	0.15986	
**ASL vs. IO**	0.06350 (871-1527)	0.11847 (211-867)	0.08216	
**Q1+Q2 vs. MEAM1**	0.13834 (486-1142)	0.19077 (841-1497)	0.16630	
**Q1+Q2 vs. IO**	0.06019 (866-1522, 871-1527)	0.09680 (191-847, … 211-867)	0.07377	
**MEAM1 vs. IO**	0.18361 (191-847 … 206-862)	0.15126 (516-1172)	0.16550	

Supplementary Table 5: Sequence divergence in the partial mtCOI sequence (708 bp) between Uganda “ASL” population and published sequences of “African-silverleafing”, “Ug4” or “okra” biotypes. The last column contains DNA and/or amino acid changes detected in comparison to reference mtCOI from Uganda “ASL” mitogenome (colour coding as in Supplementary Table 3).

Sequence	Reference	% identity to Uganda “ASL”	Sequence check
AY827606_Nigeria_IMIDA_Ni1_1	1,2	99.86	775 E->K
AY827582_Ghana_IMIDA_Gh1_4	1,2	97.87	764 L->C<br>775 E->K<br>791 E->A<br>817 Y->M<br>1415 L->S<br>1487 N->I<br>1491 K->N
AY827579_Ghana_IMIDA_Gh1_1	1,2	98.87	
AY827580_Ghana_IMIDA_Gh1_2	1,2	98.72	1250 F->C<br>1407 F->L<br>1476 E->D
AY827581_Ghana_IMIDA_Gh1_3	1,2	98.58	1250 F->C
AF344258_CamOkra	1,3	98.44	366 del T, 1357 del T, 1396 del T<br>807 L->F<br>821 A->G<br>974 L->W<br>1430 L->P
AF344285_ZIM1	1,3	99.57	366 del T, 1387 del T<br>1517 E->G
AF344286_ZIM2	1,3	99.01	366 del T, 1226 del T, 1465 del A, 1509 ins C
AY827588_okra	4	98.87	
AY827590_okra	4	98.87	
AY827587_okra	4	99.29	
AY827589_okra	4	99.01	
AY903573_Ug4_UgPkNm5	5	99.43	1003 P->A<br>1361 S->F
AY903549_Ug4_UgLeKb38	5	99.58	950 ins T
AY903551_Ug4_UgLeKb37	5	99.86	1395 I->M<br>1532 F->S
AY903531_Ug4_UgCuNm1	5	99.86	
AY903532_Ug4_UgCuNm2	5	100	
AY903545_Ug4_UgEgBkc1	5	100	
AY903546_Ug4_UgEgBkc5	5	100	
AY903571_Ug4_UgPkLu4a	5	99.72	952 del A, 1158 del T
AY903550_Ug4_UgEgNm8	5	99.86	943 ins A
AY903533_Ug4_UgCuWk22	5	100	
AY903549_Ug4_UgEgNm7	5	100	
AY903572_Ug4_UgPkLu4b	5	100	
AY903574_Ug4_UgpkNm74	5	100	
AY903534_Ug4_UgCuWk23	5	99.01	1542 del T
AY903535_Ug4_UgCuWk42	5	98.73	1083 ins C<br>908 F->Y

Supplementary Table 6: Potential synonyms of the “ASL” species used in previous publications with accessions for representative mtCOI sequences. Accessions without identified errors were included where possible; exceptions are colour-coded as in Supplementary Tables 3 and 5.

**Name**	**Reference**	**Example mtCOI sequence and reference (if different)**
Okra biotype	[4,6]	AY827589 [4]
Ug4	[5]	AY903532
J biotype	[2]	AY827589
Sub-Saharan Africa silverleafing (Q-related)	[1]	AY827579 [2]<br>AF344285 [3]
SubSaharan Africa Silverleaf	[7]	AY827606 [2]
African silverleafing (ASL)	[8]	FJ766388
Q4	[9]	AY903533 [5]
Silverleafing East and West African Mediterranean populations	[10]	FJ766391 [5]

Supplementary Table 7: Infection status of whitefly colonies used in this study by primary (P = *Portiera*) and secondary endosymbiotic bacteria (A = *Arsenophonus*, C = *Cardinium*, H = *Hamiltonella*, R = *Rickettsia*, W = *Wolbachia*). The presence and absence are indicated by plus and minus signs, respectively, reflecting the presence or absence of the PCR product after amplification with genus-specific primers.

	P	A	C	H	R	W
Spain Q1	+	-	-	+	-	-
Israel Q2	+	-	-	-	+	-
Uganda “ASL”	+	-	-	-	+	+

References

1. Boykin, L. M. *et al.* Global relationships of *Bemisia tabaci* (Hemiptera: Aleyrodidae) revealed using Bayesian analysis of mitochondrial COI DNA sequences. *Mol. Phylogenet. Evol.* **44,** 1306–1319 (2007).
2. De la Rúa, P., Simón, B., Cifuentes, D., Martinez-Mora, C. & Cenis, J. L. New insights into the mitochondrial phylogeny of the whitefly *Bemisia tabaci* (Hemiptera: Aleyrodidae) in the Mediterranean Basin. *J. Zool. Syst. Evol. Res.* **44,** 25–33 (2006).
3. Berry, S. D. *et al.* Molecular evidence for five distinct *Bemisia tabaci* (Homoptera: Aleyrodidae) geographic haplotypes associated with cassava plants in sub-Saharan Africa. *Ann. Entomol. Soc. Am.* **97,** 852–859 (2004).
4. Omondi, B. A., Sseruwagi, P., Obeng-Ofori, D., Danquah, E. Y. & Kyerematen, R. A. Mating interactions between okra and cassava biotypes of *Bemisia tabaci* (Homoptera: Aleyrodidae) on eggplant. *Int. J. Trop. Insect Sci.* **25,** 159–167 (2005).
5. Sseruwagi, P. *et al.* Genetic diversity of *Bemisia tabaci* (Gennadius) (Hemiptera: Aleyrodidae) populations and presence of the B biotype and a non-B biotype that can induce silverleaf symptoms in squash, in Uganda. *Ann. Appl. Biol.* **147,** 253–265 (2005).
6. Burban, C., Fishpool, L. D. C., Fauquet, C., Fargette, D. & Thouvenel, J. C. Host-associated biotypes within West African populations of the whitefly *Bemisia tabaci* (Genn.), (Hom., Aleyrodidae). *J. Appl. Entomol.* **113,** 416–423 (1992).
7. Dinsdale, A., Cook, L., Riginos, C., Buckley, Y. M. & De Barro, P. J. Refined global analysis of *Bemisia tabaci* (Hemiptera: Sternorrhyncha: Aleyrodoidea: Aleyrodidae) mitochondrial cytochrome oxidase 1 to identify species level genetic boundaries. *Ann. Entomol. Soc. Am.* **103,** 196–208 (2010).
8. Gueguen, G. *et al.* Endosymbiont metacommunities, mtDNA diversity and the evolution of the *Bemisia tabaci* (Hemiptera: Aleyrodidae) species complex. *Mol. Ecol.* **19,** 4365–4376 (2010).
9. Chu, D. *et al.* Use of mitochondrial cytochrome oxidase I polymerase chain reaction-restriction fragment length polymorphism for identifying subclades of *Bemisia tabaci* Mediterranean group. *J. Econ. Entomol.* **105,** 242–251 (2012).
10. De Barro, P. J. The *Bemisia tabaci* species complex: Questions to guide future research. *J. Integr. Agric.* **11,** 187–196 (2012).
